# Early Marriage, Cohabitation, and Childbearing in West Africa

**DOI:** 10.1155/2019/9731756

**Published:** 2019-06-13

**Authors:** Winfred A. Avogo, Oluwaseyi D. Somefun

**Affiliations:** ^1^Illinois State University, Department of Sociology and Anthropology, Campus Box 4660, Normal, IL 61790-4660, USA; ^2^University of the Witwatersrand, Demography and Population Studies, Johannesburg, South Africa

## Abstract

The prevalence of child marriage in West Africa is one of the highest in the global south. Yet, much of what we know about the harmful effects of early marriage and why it persists comes from research on South Asia. Adopting life course family development perspectives on adolescent sexuality, we examine the linkages between the timing of union formation and childbearing across multiple countries with high rates of child marriage. Using the latest round of data from the Demographic and Health Surveys (DHS), we find that by age 18, 28 percent of adolescents in Nigeria, 25 percent in Burkina Faso, and as high as 60 percent in Niger are in a union, whilst 13 percent of Nigerian adolescents, 12 percent in Burkina Faso, and 27 percent in Niger have had a first birth. The results demonstrate that, net of individual characteristics, community variables are strong predictors of union formation and childbearing. Individual characteristics such as women's education, economic status of households, and residing in female-headed households and rural areas are other salient determinants of adolescent family transitions. We discuss the findings in the context of revamping stalled fertility transitions and the post-2015 framework for development in sub-Saharan Africa.

## 1. Introduction

Fertility transition is now a global phenomenon. With the exception of a few sub-Saharan African (SSA) countries, fertility decline is well underway in almost all other regions of the world. The key drivers of this transition are macrolevel structural and economic changes and microlevel cultural value orientations that occur independently or simultaneously. The impact of macrolevel changes is evident in SSA where changes in fertility and family size have been closely linked with economic changes. However, in recent years, the lack of progress in socioeconomic development may be partly responsible for the slow pace of fertility decline in SSA [1–3].

A closely linked aspect of fertility transition is changes in union formation. Although there has been much progress in increasing age at marriage worldwide, 21 percent of young women (aged 20 and 24) still marry before the age of 18. South Asia and SSA remain home to the largest number of child brides (44 percent and 18 percent, respectively) [4]. However, within West and Central Africa, the regions with the highest prevalence of child marriage in the world, evidence of a decline in age at marriage has stalled in many countries (Niger, Mali, and Burkina Faso) and in some countries (Nigeria and Niger) early marriage rates appear to be increasing [5]. Yet, despite the high prevalence of early marriage in West Africa, the sociocultural and socioeconomic determinants have not been extensively studied. Much of what we know come from research on South Asia [6, 7]. Further research is needed to understand the underlying structural, cultural and individual motivations that promote early marriage in the region. This would aid in policy formulation to promote later marriage which in turn could have implications for fertility transitions in the region.

In this paper, we use data from the Demographic and Health Surveys to first present nationally representative estimates of the prevalence, trends and variations in union formation and childbearing among women 15–18 years in three countries (Nigeria, Burkina Faso, and Niger) in West Africa. Then, we examine the relationship between individual, household, and community-level characteristics on the timing of early union formation and childbearing. We interpret the results within the broader context of adolescent life course family development trajectories in a region plagued by poverty, conflict, and with resource-rich economies that continue to grow but with staggering levels of youth unemployment thereby dwindling the chances of a conventional fertility transition.

## 2. Background and Literature Review

Child marriage—defined by the United Nations as marriage or cohabitation before the age of 18—is a human rights violation. It undermines girls' right to autonomy, development, and the attainment of public health goals. According to the Human Rights Commission, these rights must be universal and guaranteed by the state, inalienable and cannot be taken away, indivisible, interrelated, and interdependent [8]. Although child marriage is not specifically mentioned in the 1989 United Nations Convention on the Rights of the Child (CRC), several provisions of this convention are violated by the practice. For example, Article 6 of CRC provides that “State parties shall ensure to the maximum extent possible the survival and development of the child.” Since child marriage is followed by expectations of childbirth, it has grave consequences for both the young mother and her child. Research has shown that early pregnancy is consistently linked to increased risk of maternal and infant morbidity and mortality [9].

Similarly, the 1990 African Charter on the Rights and Welfare of the Child (ACRWC) which has been ratified by all African countries (except thirteen of them) mandates that African countries should take action to end child marriage. Article 21 of the Charter states that “Governments should do what they can to stop harmful social and cultural practices, such as child marriage, that affect the welfare and dignity of children” [10]. However, the political will to change and implement laws that will end child marriage are at best inconsistent and in some cases, fall far short to protect the dignity and welfare of the child. Economic factors, social, cultural, and religious influences contribute to the persistence of early marriage in the region. Moreover, due to population growth, SSA is on track to surpass South Asia as the region with the highest number of girls married as children [4].

Yet much of what we know about adolescent cohabitation and marriage in developing settings is mostly from research conducted in South Asia [7]. For example, a study concerning India, Bangladesh, Nepal, and Pakistan found an association between child marriage and nonuse of contraception before the first birth, rapid repeated childbirth, miscarriages, and stillbirths, as well as unintended pregnancies and abortion [11]. Similarly, child brides were less likely to receive proper medical care during pregnancy and were least likely to have delivered their most recent baby in a health facility. They also had fewer years of schooling, lower labor force participation and overall weak economic prospects [6, 12]. These negative outcomes were, unsurprisingly, more severe among women who married in their early teen years (below age 15) compared to those who married in middle adolescence or thereafter [5, 7, 13].

However, a new body of research is emerging on the consequences of early marriage in SSA. Much of that research has focused on risk factors for HIV/AIDS [14–17] and finds that younger brides have higher rates of HIV infection than their unmarried sexually active peers [18] likely due to greater age disparity with partners who may already be infected and their inability to negotiate safe sex [16]. Other research on the disproportionate burden posed by early marriage and childbearing on reproductive health problems in SSA finds that the risk of early marriage increases with lower levels of education. Nearly two-thirds of women with no formal education in Malawi were child brides compared to one-third among women who had a formal education [19]. Another study on the role of child marriage and childbirth among school dropouts in Francophone Africa found that early marriage limits girls' education more than early childbirth or “school girl pregnancy” [20].

What is needed is a rigorous expansion of the empirical underpinnings of the links between early marriage, childbirth, and its sociocultural determinants in West Africa, which has simultaneously the world's worst prevalence of child marriage as well as its highest fertility. More than half of women born between the ages of 20–24 in four West African countries (Burkina Faso, Guinea, Mali, and Niger) married before age 18 [5]. Most of these brides are likely to be married at a very early age of 9 to 12, the earliest in the developing world [21]. In Niger, for example, the country with the highest overall prevalence of child marriage, 77 percent of women aged 20–24 were married before age 18. In Nigeria, although its prevalence rate is much lower than Niger, it has the highest absolute number of married girls than in all the countries in the region put together.

Thus using adolescent life course family development perspectives, we examine the relationship between the timing of union formation and childbearing and their sociocultural determinants among young women aged 15–19 across three countries—Nigeria, Burkina Faso, and Niger—in West Africa using the latest round of data from the Demographic and Health Surveys (DHS).

## 3. Conceptual Framework and Hypotheses

The theoretical framework of this paper draws from *life course family development perspectives*. These perspectives have been used variously by psychologists (individual life span theory), sociologists of the family (family development theory), and demographers (life course theory) to explain the changing form and structural development of the individual in the context of a family and how family experiences overtime influence later life outcomes such as cohabitation, marriage, fertility, and divorce [22]. In addition, these perspectives are relevant in examining the changing roles of the family within the broader structural context of society—political, economic, social, cultural, community, and environmental conditions—that affect the health and well-being of adolescents. Thus this framework is appropriate for investigating how macrolevel social changes (the community context) affect microlevel behavior (such as marriage and fertility) [23]. However, adolescence as a key life course developmental stage, especially in the social context of SSA, is least explored.

Cohabitation, marriage, and parenthood are very stressful events in the lives of adolescents and their families particularly in West Africa where poverty levels are high, unemployment is rampant, quality education (especially secondary education) is unaffordable, political conflict is rife and rapid climate change leads to erratic rainfall, food shortages, and desertification. In these dire circumstances, parents often regard large numbers of children as an economic burden, adolescent girls are viewed as a potential source of dowry income, and the status of women is low [21]. Although, at the aggregate level, these factors are dismal for many families, and they may conceal neighbourhood and community-level factors that can reduce early union formation and childbearing among adolescents. Applying life course family development perspectives will allow an investigation of how macrolevel social changes or community characteristics influence microlevel sexual behavior of adolescents.

We argue that adverse reproductive health outcomes of adolescents (such as early marriage and childbirth) develop overtime within an individual life course, in the family, community, across generations, and at different population levels. Health outcomes of adolescents correspond strongly from birth, their upbringing within a household, and the levels of opportunity provided by a nation as young people grow into adult life. Women who marry early are more likely to have mothers who married early or are single mothers. They may also come from disadvantaged backgrounds in terms of income inequality and limited access to primary and secondary education [24]. They are most likely to lack safe and supportive families, schools, peers, and communities to help them avoid risks and realize their full potential [25].

Thus we hypothesize that due to strong cultural traditions and the low status of women in West Africa, neighbourhood characteristics such as the proportion of educated women, the level of poverty, and working women in the community will be significantly associated with union formation and first childbearing. Also, individual characteristics such as education and religion and family-level characteristics such as household wealth, the sex of the household head, the region of residence, and rural/urban locations will be significantly associated with union formation and first childbearing.

## 4. Data and Methods

Data for the analyses that follow come from the Demographic and Health Surveys (DHS) of three countries in West Africa (Nigeria, Niger, and Burkina Faso) with the highest prevalence and absolute number of child brides in the region. The surveys were conducted in 2013 for Nigeria, 2012 for Niger, and 2014 in Burkina Faso. The DHS used a two-stage sample design. In the first stage, stratified sampling techniques were used to select clusters as the primary sampling unit. The second stage involved a systematic sampling of households within each cluster. The current sample is based on 6653 females in Nigeria, 2690 in Burkina Faso, and 1570 in Niger who are aged 15–18 and who responded to questions related to the start dates of first union and first birth. Using reports from younger women on age at marriage reduces bias compared to older women who often report events closer to the time of the survey than they actually happened [5, 26].

Since the DHS sample is not self-weighting at the national and subsample levels, weighting factors (provided in the data) are used to produce multivariate results that are proportional at the national level and account for unequal probability of selection. Individual weights were used for descriptive statistics.

## 5. Variable Measurement

### 5.1. Dependent Variables

Two dependent variables form the basis of the analysis: the timing of respondent's first union (defined in the DHS as the start date of cohabitation and marriage) and the timing of the first live birth. For the first dependent variable, the DHS questionnaire asked women if they had been married or had lived with a man only once or more than once. For women who had been married or had lived with a man only once, they were asked to provide the month and year they started living with their husband/partner, and for women who had been married or had lived with a man more than once, they were asked the month and year they started living with the first husband/partner. The analysis is therefore based on the year of the start of first union. For the second dependent variable, women were asked the month and year of each of their births, whether still alive or not; starting with their first birth. Like first union, this analysis is also based on the year of their first birth. Both outcomes were coded as time-varying for dynamic modelling using event history techniques.

### 5.2. Independent Variables

Guided by research that points to neighbourhood context or the effect of the community factors as determinants of reproductive behavior [27], we use three community variables as key independent variables to capture the neighbourhood context. These include the percentage of educated women in the community, the percentage of poor women, and the percentage of working women. These variables are constructed by aggregating the individual and household-level variables at the primary sampling unit (PSU) which served as a proxy for community-level variables.

### 5.3. Covariates

The survey collected information on the characteristics of respondents at the time of the survey. We include variables on the place of residence (urban or rural), region of residence, religion, the wealth index of the household, educational attainment, and the sex of the head of the household as a proxy for women's empowerment. All the covariates are fixed or are time-invariant. Although all the covariates were based on information on the current characteristics of respondents and the dependent outcomes are time-varying, we assume that these characteristics are less likely to be influenced by any particular changes affecting adolescents. We also include a variable for the duration/time, i.e., years of risk from age 10 to marriage or cohabitation or interview if censored.

## 6. Analytical Methods

Standard survival analyses and discrete-time logistical regression models are used. As indicated, two events are of interest: whether a woman is in union or not and whether a woman has had a first birth. The risk of both events begins from the age of ten since child marriage is fairly common in West Africa. For women who experience both events, the risk begins from the time they reach the age of ten to the year of both events. Women who did not experience the events are censored at the year of interview. The duration of risk is measured in years. The discrete-time model is based on person-year risk of the events and is estimated using a logistic function and time-varying predictors [28, 29]. All analyses are conducted in SAS using multilevel models with random effects procedures to account for the sampling design and to produce adjusted estimates (odds ratios) and standard errors [30].

## 7. Results

### 7.1. Descriptive Characteristics of Respondents

**Figure 1:**
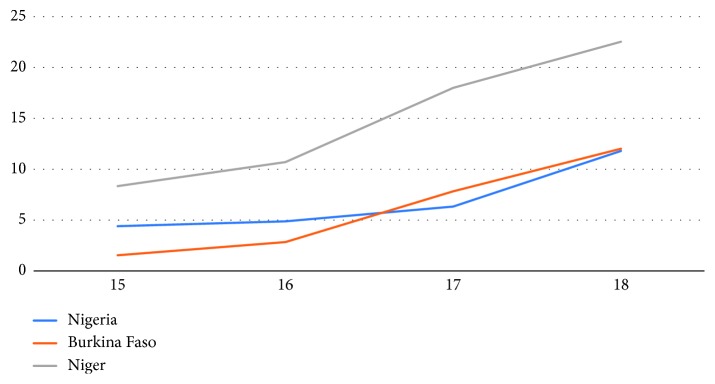
Percent of union formation by respondent's age.

Figure 1 shows the percent distribution of adolescents aged 15–18 who are cohabitating or married (union formation) in the three countries analysed. The figure shows that, in all three countries, union formation increases steadily with age. By age 18, 27.41 percent of adolescents are in union in Nigeria, 24.23 percent in Burkina Faso, and nearly 60 percent in Niger.

**Figure 2:**
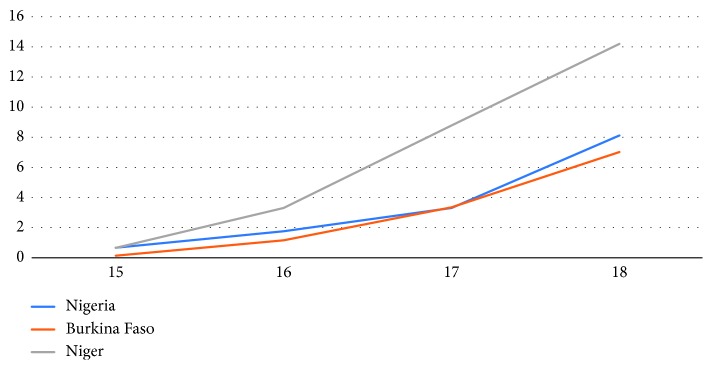
Percent of first childbearing by respondent's age.

Figure 2 presents similar patterns for first childbearing among adolescents. By age 18, 13 percent of Nigerian adolescents, 12 percent in Burkina Faso, and 27 percent in Niger have had a first birth. There are slightly more adolescents in union who have not started childbearing (15 percent in Nigeria, 14 percent in Burkina Faso, and 33 percent in Niger) than those in union who have had a first birth (13 percent in Nigeria, 10 percent in Burkina Faso, and 27 percent in Niger). Single adolescent mothers were rare in all three countries (less than 2 percent) (results not shown).

Table 1 presents bivariate analysis of the study samples in all three countries. All the socioeconomic characteristics in all three countries were significantly associated with both outcomes given the chi-square statistic. Union formation and childbearing status differed by education level. Adolescent girls with no education were more likely to form a union or have a first birth than those with primary and secondary or higher education. Similarly, adolescent girls in the richest quintile of the household wealth index were less likely to be in union or to have a first birth. Muslim girls formed unions (89 percent and 77 percent) and had their first births (79 percent and 71) at higher proportions in Nigeria and Burkina Faso than girls who were Christian or of other religion (religion was not measured in Niger). Adolescent girls who reside in the Southern part of Nigeria had lower rates of union formation and childbearing than in Northern Nigeria. However, in Burkina Faso, the Southern part of the country had higher rates of both outcomes than the Northern part. Lastly, adolescents who reside in female-headed households had lower rates of both outcomes than in male-headed households. However, the proportion of first childbearing in female-headed households was higher than that of union formation. Both outcomes were also common in rural than in urban areas.

### 7.2. Multivariate Findings (Odds Ratios)

Table 2 shows adjusted odds ratios of four models predicting *union formation* among adolescents in Nigeria, Burkina Faso, and Niger. The first model includes variables measured at the community level to test neighbourhood effects, and the second model is the full model that includes all predictors.

It can be observed from Model 1 that community-level characteristics mostly have a statistically significant influence on union formation. A medium to high proportion of educated women in a community decreases the risk of union formation in all three countries. For example, in Niger, the odds of union formation of a woman who lives in a community with a higher proportion of educated women are 0.343 times lower compared to those of a woman who lives in a community with a low level of educated women (the odds in Nigeria and Burkina Faso are 0.068 and 0.278, respectively). Similarly, the level of community poverty increased the odds of union formation in all countries. A woman in Nigeria who lives in a community with a high level of household poverty is nearly 4 times (3.657) more likely to form a union than the one who lives in a community with a low level of poverty (the odds are 1.6 and 1.9 times in Burkina Faso and Niger, respectively, but are not statistically significant). Lastly, apart from Nigeria where a high proportion of working women in a community reduced the odds of union formation (0.565), we did not find any significant associations between the proportion of women working in the community and union formation in Burkina Faso and Niger.

In Model 2 the full model, which includes community, individual, and household characteristics, the direction and magnitude of the community variables have not changed as much from Model 1. After controlling for all variables, a medium or high proportion of educated women in the community significantly decreases the risk of union formation in Nigeria (0.438) and Burkina Faso (0.735) but not in Niger. Similar results are obtained for a high percentage of poor women in a community, which increases the odds of union formation by 4.482 times in Nigeria but not in Niger (although *p* < 0.1) and Burkina Faso. The last community variable, and the proportion of working women was not found to be statistically significant in all three countries.

On individual characteristics that influence union formation, women's level of education at both the primary and secondary levels significantly reduces the risk of union formation in all countries. The odds of union formation for a woman with a secondary education are 0.162 less in Nigeria, 0.259 in Burkina Faso, and 0.226 in Niger. At the household level, the wealth index, as a measure of socioeconomic status, increases the risk of union formation. For example, women from the richest quintile of the wealth index in Nigeria are two times (2.322) more likely to form a union compared to those in the poorest quintile. The magnitude of the odds although lower at the poorest quintile is nonetheless statistically significant in Nigeria (1.547). Christianity reduces the risk of union formation in Nigeria but increases the risk in Burkina Faso compared to Muslim and other religions (religion as not measured in Niger, where more than 90 percent of the population is Muslim). Living in the southern part of Nigeria also reduces the risk of union formation but not in Burkina Faso and Niger. Residing in a female-headed household significantly reduces the risk of union formation in Nigeria and Burkina Faso but not in Niger. Rural residence is only significant in Niger where the odds of union formation among rural adolescent women are nearly 3 times that of their urban counterparts.

Next, we summarize the results of *first childbearing status* among adolescents in Table 3.

Community-level variables were generally not statistically significant in the full model (Table 3 Model 2 in Burkina Faso and Niger). However, in Nigeria, the proportion of educated women in the neighbourhood reduces the risk of childbearing. The odds of first birth for women living in a community with a medium level of education were 0.551 less than women who live in communities with a low level of education. Although, some community-level variables were significant in model 1 for Nigeria, they were not found statistically significant in Model 2.

On individual characteristics, women's education at the secondary or higher level significantly reduces the risk of childbearing in Nigeria and Burkina Faso but not for Niger. Similarly, in Nigeria, household wealth decreases the risk of first birth at all quintiles compared to the poorest quintile. Household wealth was however, not statistically significant in Burkina Faso and Niger. Religion increases the risk of birth in Nigeria but not in Burkina Faso. Living in the south of Nigeria also increases the risk of first birth for adolescent females but not in Burkina Faso and Niger. Finally, contrary to the model on union formation (Table 2) living in a female-headed household increases the risk of childbearing in Nigeria and not in the other two countries, whilst rural residence increases the risk in Nigeria. Rural residence was not statistically significant in Burkina Faso and Niger.

## 8. Discussion and Conclusion

Overall, the results confirm that union formation and first childbearing of adolescent girls in West Africa is high, especially in Niger where the rates of both outcomes are more than double that of Nigeria and Burkina Faso. The multivariate results show that community characteristics are significant predictors of adolescent transitions (union formation and childbearing) especially in Nigeria. On individual-level characteristics, adolescent girl's education decreases the risk of union formation in all countries examined, whilst on childbearing, education decreases the risk in Nigeria and Burkina Faso. The results of other socioeconomic characteristics are similar; household wealth increases the risk of union formation but decreases the risk of first birth especially in Nigeria. Christianity lowers the risk of union formation and increases the risk of childbearing. Residing in southern parts of the countries and living in female-headed households decreases the risk of union formation and increases the risk of childbearing (especially in Nigeria). Finally, rural residence is associated with an increase in risk on both outcomes (in Niger and Nigeria).

These results demonstrate the importance of considering both the macro-sociocultural context and microindividual-level influences on adolescent sexual and reproductive behaviors. The association of community-level variables with union formation and childbearing showed that neighbourhood effects are important to be considered. The effect of neighbourhood factors on the health and behaviors of young people correspond strongly across the life course, from childhood to adult life, and are crucial for the health and economic development of a community. Consistent with previous research, community characteristics have an effect on the reproductive behavior [31]. However, this has largely been demonstrated for limiting fertility—e.g., contraceptive use—and for infant and child mortality [32] than for early marriage.

The finding that individual-level characteristics such as education and religion and household characteristics such as the wealth status of a household, the sex of the household head, and your place of residence are consistent with previous literature. Previous research has found that primary schooling of girls did not necessarily raise the age at marriage as union formation follows after primary school completion or drop out [21] thereby curtailing educational attainment and compromising labor force participation. Low levels of education of adolescent girls are associated with increased risk of repeated childbearing, shorter birth intervals, and higher overall fertility over the life course [8]. After marriage, adolescent girls usually experience pressure from their older partners and their extended family to prove their fertility and have a child [33]. This increases the risk of morbidly and mortality for them and their children, given their physiological immaturity [5]. Unintended births and repeated childbearing also shortens women's educational attainment and undermines their economic potential.

Most girls are expected to move into their partner's family household and become the responsibility of their in-laws. Indeed, much of the motivation for child marriage is to secure the financial security of the child bride and to decrease the economic burden of caring for girls [34]. Marriage often attracts a dowry for the bride's family, and the younger the girl is, the higher the bride price. Moreover, payment of dowry is usually required for a girl who has given birth to be reunited with her partner and his family. Thus in West Africa, there is a real economic incentive for early marriage, given the dire economic circumstances and strong cultural traditions in the region.

A few recommendations arise from this study, and research on the negative effects of adolescent family transitions must consider the early life course of children as it forms the foundation for later family transitions. Health interventions for adolescents must account for factors that improve the conditions of daily living (education and employment opportunities) across the life course and address risk factors and stressors within the community, in the family and individual social lives of young people. Young people need safe and supportive families, schools, and communities to thrive and realize their full potential. Adolescence as a key life course developmental stage must be fully incorporated into all the new SDGs on SSA, as its long-term implications for the health and economic development of the entire region is at stake. Such goals must refocus efforts in SSA on structural changes to improve access to education, employment, and recreational opportunities and to improve the conditions of young females in rural communities.

Family planning programs must be revamped and the political will to implement them and overcome the numerous barriers (such as psychological barriers—embarrassment and fear—confidentiality, and financial constrains) that young people face in accessing reproductive health services must be addressed [35]. As a specific target for achieving goal number five of the SDGs, eliminating child marriage must be part of family planning campaigns to accelerate the attainment of that goal by 2030.

Finally, this study is not without some limitations. The cross-sectional nature of the data and the limited number of covariates and predictors included, especially given that they are not all time-varying and measured prior to the events of interest, limits the conclusions of this study. More complete retrospective data are needed to allow greater understanding of the dynamics of adolescent sexual and reproductive health transitions. In addition, although caution was taken to ensure that the key predictors of the study (union formation and childbearing) are time-varying, and to limit age measurement bias, concerns remain that cohabitation, marriage, and childbearing are joint processes, especially in SSA and are more complex than explored in this study. Similarly, our data are unable to distinguish between transitions into cohabitation separately from those into marriage as the two are measured together. Nonetheless, the results not only make a useful contribution to understanding the trajectories of family life for young people but move away from descriptive assessments to consider age variations in family life course experiences, and the community context within which these experiences occur in West Africa.

## Figures and Tables

**Table 1:** Bivariate distribution of union formation and first childbearing status by socioeconomic characteristics in West Africa.

Variable	Nigeria		Burkina Faso		Niger	
	Union formation	Childbearing	Union formation	Childbearing	Union formation	Childbearing
Respondent's education						
None	73.93	67	80.73	77.82	79.45	81.61
Primary	13.65	14.7	12.4	16.56	14.36	10.77
Secondary/higher	12.43	18.31	6.87	5.62	6.1	7.61

Wealth index						
Poorest	40	37.21	20.82	20.64	18.58	20.69
Poorer	33.8	33.14	24.99	23.28	21.85	23.81
Middle	15.87	17.4	21.49	22.21	23.29	21.06
Richer	7.76	8.71	18.61	19.94	23.37	21.93
Richest	2.56	3.53	14.08	13.93	12.91	12.51

Religion						
Muslim	89.01	79.21	76.87	71.1	—	—
Christian	9.96	19.8	14.98	19.12		
Other	0.53	0.19	8.15	9.78	—	—

Region						
Northern	94.62	85.55	34.17	32.82	98.94	98.26
Southern	5.38	14.45	65.83	67.18	1.06	1.74

Sex of household health						
Male	95.29	90.7	93.88	90.42	89.13	85.41
Female	4.71	9.3	6.12	9.58	10.87	14.59

Place of residence						
Urban	13.25	14.37	15.97	17.42	7.23	7.5
Rural	86.75	85.63	84.03	82.58	92.77	92.5

Percent	27.41	13.85	24.24	11.68	59.66	26.95
Total	6653	6653	2690	2690	1570	1570

All the relationships were found statistically significant using the chi-square values.

**Table 2:** Odds ratios of discrete-time logistic regression estimates of union formation among adolescents in Nigeria (*N*=6653), Niger (*N*=1570), and Burkina Faso (2690).

Variables	Model 1			Model 2		
	Nigeria	Burkina Faso	Niger	Nigeria	Burkina Faso	Niger
Community-level variables						
Percentage of women educated						
Low	1	1	1	1	1	1
Medium	0.206^*∗∗∗*^	0.528^*∗∗∗*^	0.901	0.681^+^	0.735^*∗*^	1.191
High	0.068^*∗∗∗*^	0.278^*∗∗∗*^	0.343^*∗∗∗*^	0.438^*∗∗*^	0.838	0.947

Percentage of poor women						
Low	1	1		1	1	1
Medium	1.827^*∗∗*^	1.025	2.343^*∗*^	1.812^+^	0.885	1.387
High	3.657^*∗∗∗*^	1.578^*∗*^	1.917	4.482^*∗∗∗*^	1.543^+^	1.307

Percentage of working women						
Low	1	1		1		1
Medium	0.785	0.932	1.174	1.007	0.925	1.129
High	0.565^*∗∗*^	0.81	0.735	0.787	0.863	0.724

Respondent's education						
None				1	1	1
Primary				0.447^*∗∗∗*^	0.384^*∗∗∗*^	0.494^*∗∗∗*^
Secondary/higher				0.162^*∗∗∗*^	0.259^*∗∗∗*^	0.226^*∗∗∗*^

Wealth index						
Poorest				1	1	1
Poorer				1.547^*∗∗*^	1.357^+^	1.327
Middle				2.173^*∗∗∗*^	1.358^+^	1.509
Richer				3.123^*∗∗∗*^	1.58^*∗*^	1.749^+^
Richest				2.322	0.954	2.022^+^

Religion						
Muslim				1	1	1
Christian				0.642^*∗∗*^	1.989^*∗∗∗*^	-

Region						
Northern				1	1	1
Southern				0.522^*∗∗*^	0.929	1.2

Sex of household health						
Male				1	1	1
Female				0.591^*∗∗∗*^	0.596^*∗*^	0.696

Place of residence						
Urban				1	1	1
Rural				1.268	1.412	3.272^*∗∗∗*^

*N* (person-years)	50169	19918	11426	50169	19918	11426

^*∗*^=*p* < 0.05, ^*∗∗*^=*p* < 0.01, ^*∗∗∗*^=*p* < 0.001, ^*+*^=*p* < 1.0.

**Table 3:** Odds ratios of discrete-time logistic regression estimates of first childbearing among adolescents in Nigeria (*N*=6653), Niger (*N*=1570), and Burkina Faso (2690).

Variables	Model 1			Model 2		
	Nigeria	Burkina Faso	Niger	Nigeria	Burkina Faso	Niger
Community-level variables						
Percentage of women educated						
Low	1	1	1	1	1	1
Medium	1.068	1.411	0.775	0.551^*∗*^	1.526	0.859
High	1.165	1.563	0.918	0.514^+^	1.766	1.218

Percentage of poor women						
Low	1	1		1	1	1
Medium	2.643^*∗∗∗*^	0.836	0.885	1.383	0.807	0.707
High	2.313^*∗∗*^	0.696	1.08	0.62	0.744	0.764

Percentage of working women						
Low	1	1		1		1
Medium	1.945^*∗*^	1.233	1.95^+^	1.35	1.225	1.967^+^
High	2.609^*∗∗*^	1.121	1.456	1.676^+^	1.106	1.553

Respondent's education						
None				1	1	1
Primary				0.875	1.751^+^	0.975
Secondary/higher				0.396^*∗∗*^	0.354^*∗*^	0.466

Wealth index						
Poorest				1	1	1
Poorer				0.491^*∗∗*^	2.841	0.858
Middle				0.371^*∗∗*^	2.387	0.932
Richer				0.304^*∗∗∗*^	1.682	0.618
Richest				0.239^*∗∗∗*^	1.083	0.569

Religion						
Muslim				1	1	1
Christian				4.357^*∗∗∗*^	1.046	

Region						
Northern				1	1	1
Southern				2.462^*∗∗∗*^	1.182	0.461

Sex of household health						
Male				1	1	1
Female				1.711^*∗*^	1.021	1.007

Place of residence						
Urban				1	1	1
Rural				2.722^*∗∗∗*^	0.543	0.955

*N* (person-years)	50169	19918	11426	50169	19918	11426

^*∗*^=*p* < 0.05, ^*∗∗*^=*p* < 0.01, ^*∗∗∗*^=*p* < 0.001, ^*+*^=*p* < 1.0.

## Data Availability

Data from the Demographic and Health Surveys (DHS) can be obtained free of charge from https://dhsprogram.com/data/.
